# *Lonomia obliqua* Envenoming and Innovative Research

**DOI:** 10.3390/toxins13120832

**Published:** 2021-11-23

**Authors:** Miryam Paola Alvarez-Flores, Renata Nascimento Gomes, Dilza Trevisan-Silva, Douglas Souza Oliveira, Isabel de Fátima Correia Batista, Marcus Vinicius Buri, Angela Maria Alvarez, Carlos DeOcesano-Pereira, Marcelo Medina de Souza, Ana Marisa Chudzinski-Tavassi

**Affiliations:** 1 Centre of Excellence in New Target Discovery (CENTD), Butantan Institute, Butantã 05503-900, SP, Brazil; miryam.flores@butantan.gov.br (M.P.A.-F.); renata.gomes@esib.butantan.gov.br (R.N.G.); dilza.silva@butantan.gov.br (D.T.-S.); douglas.oliveira@butantan.gov.br (D.S.O.); isabel.batista@butantan.gov.br (I.d.F.C.B.); marcus.buri@butantan.gov.br (M.V.B.); angela.alvarez@esib.butantan.gov.br (A.M.A.); carlos.ocesano@butantan.gov.br (C.D.-P.); marcelo.souza@butantan.gov.br (M.M.d.S.); 2 Development and Innovation Centre, Butantan Institute, Butantã 05503-900, SP, Brazil

**Keywords:** *Lonomia*, envenoming, innovation

## Abstract

As a tribute to Butantan Institute in its 120th anniversary, this review describes some of the scientific research efforts carried out in the study of *Lonomia* envenoming in Brazil, a country where accidents with caterpillars reach over 42,000 individuals per year (especially in South and Southeast Brazil). Thus, the promising data regarding the studies with *Lonomia*’s toxins contributed to the creation of new research centers specialized in toxinology based at Butantan Institute, as well as to the production of the antilonomic serum (ALS), actions which are in line with the Butantan Institute mission “to research, develop, manufacture, and provide products and services for the health of the population”. In addition, the study of the components of the *Lonomia obliqua* bristle extract led to the discovery of new molecules with peculiar properties, opening a field of knowledge that could lead to the development and innovation of new drugs aimed at cell regeneration and inflammatory diseases.

## 1. Introduction

Although lepidopteran species are widely distributed around the world, only a few of them cause severe damage to humans or animals that have had contact with adult animal hairs (lepidopterism) or with the bristles of caterpillars (erucism) [1]. Locally, accidental contact with hair or bristles leads to a skin reaction, and systemic symptoms can be treated using oral antipruritic and antihistamines [2]. However, some caterpillar species of the *Lonomia* genus cause serious injuries, which are sometimes irreversible, leading to death. Patients that develop clinical manifestations of disseminated intravascular coagulation (DIC) and consumptive coagulopathy can progress to hemorrhagic syndrome with serious consequences if the antilonomic serum (ALS) produced by the Butantan Institute (SP/Brazil) is not administered in due time [3,4,5,6,7,8]. Although treatment with ALS is effective for *Lonomia*’s envenoming, deaths resulting from contact with caterpillars are still a public health problem in Brazil [9,10]. The literature reports that, between 2007 and 2017 a total of 42,264 accidents were caused by caterpillars in Brazil, among them 248 were severe cases and five evolved to deaths. Most accidents occurred in the states of south and southern Brazil between December and April, a period corresponding to an increase in temperature and rainfall [10].

**Figure 1:**
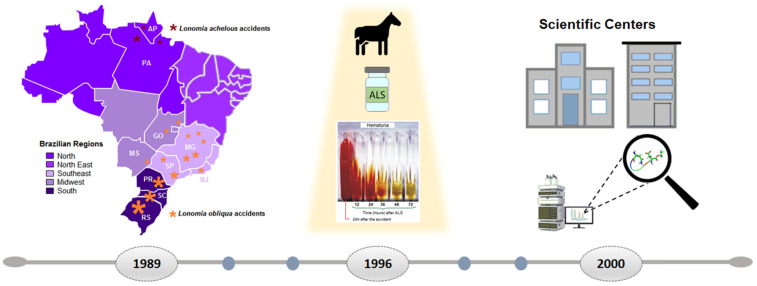
Overview of *Lonomia obliqua* epidemiology, treatment, and research over the years. Since 1989, a burst of accidents with hemorrhagic manifestations were reported in Brazil to be caused by *L. obliqua* (Walker, 1855) (orange asterisks), mainly in Santa Catarina (SC), Rio Grande do Sul (RS), and Paraná (PR). In Venezuela and northern Brazil [Amapá (AP) and Pará (PA)], caterpillars were identified as *L. achelous* (Cramer) (red asterisks). Other cases were registered in the states of Goiás (GO), Minas Gerais (MG), São Paulo (SP), Mato Grosso do Sul (MS), and Rio de Janeiro (RJ) (orange asterisks). In 1996, an antivenom against *L. obliqua* toxins was developed [3]. Today, the treatment of patients is based on the administration of ALS, produced at the Butantan Institute, which has been shown to be effective in reversing hemostatic and hemorrhagic disorders. Photograph (Dr. Marlene Zannin) showing the reduction in hematuria of urine samples of a patient with treatment started with ALS after 24 h of having an accident with *L. obliqua* caterpillars. In 2000, the State of São Paulo Research Foundation (FAPESP) started a program to create Research, Innovation, and Dissemination Centers (RIDC) leading to the creation of the “Center for Applied Toxinology (CAT)”, the “Center of Toxins, Immune Response, and Cell Signaling (CETICS)”, and the “Center of Excellence in New Target Discovery (CENTD)”, with the latter aiming at not only the study of toxins from poisons and animal secretions, but also the development of new molecules based on toxins and, in public–private partnerships, their use as tools for studying molecular targets for several diseases.

Over the years, Brazil has gained significant knowledge in the field of toxinology that benefits the politics of public health. One example is the existence of Toxicological Information and Assistance Centers (CIATox), created for the Brazilian Unified Health System (SUS) to provide specific information on poisoning and treatment to health professionals and to the community. Furthermore, the creation of special programs and centers for research in the study of animal toxins contributed to the innovation in the development of new molecules derived from animal toxins or secretions, accelerating the interaction between science and industry. Therefore, this review highlights the current knowledge about *Lonomia* envenoming, as well as its treatment and already identified bioactive molecules, approaching the future perspectives on innovative research with new derived compounds as potential drugs for the treatment of inflammatory diseases (Figure 1).

## 2. *Lonomia* spp. Epidemiology and Impact on Public Health

**Figure 2:**
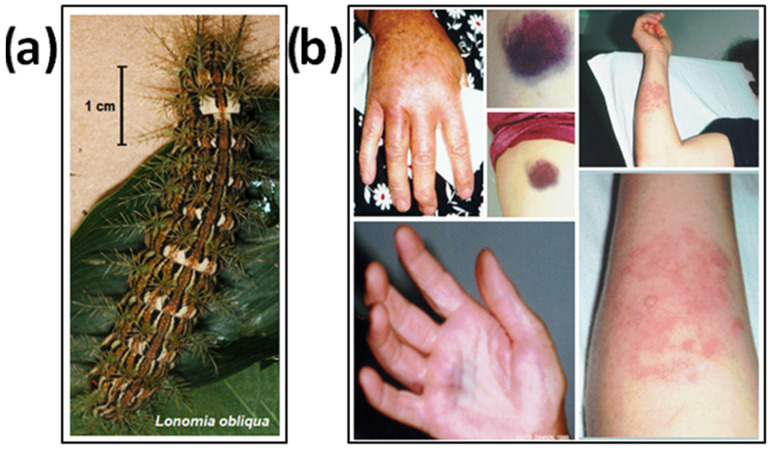
*L. obliqua* and clinical manifestations. (**a**) *L. obliqua* caterpillar. This photograph shows a caterpillar at the sixth stage or instar; and (**b**) Initial symptoms. This photograph shows some clinical manifestation that begin 12 to 24 h after the accident involving contact with broken bristles. Edema (hands), erythema, heat, and blisters (arm), in addition to systemic symptoms, have been reported. Ecchymosis, after 3 days of contact, of variable intensity and hematuria (abdominal bruises, after 24 h), may occur (Photographs: (**a**) Dr. Miryam P. Alvarez-Flores; (**b**) Dr. Marlene Zannin).

The occurrence of hemorrhage after contact with South American caterpillars was first reported by Alvarenga and collaborators [11]. Although 26 species of the genus *Lonomia* (Saturniidae family) are distributed in the American continent, the most studied species are *L. obliqua* (Figure 2a) and *Lonomia achelous* caterpillars; both are capable of inducing hemorrhagic effects in humans after contact with their broken spines [8,12,13,14,15,16,17,18,19,20].

Accidents involving *L. achelous* caterpillars have been reported in Venezuela since 1967 [21] and in Brazil since 1982, in the state of Amapá and Ilha de Marajó (state of Pará) [15,22]. Accidents related to *L. achelous* have been characterized by a hemorrhagic syndrome attributed to the fibrinolytic activity of the venom [23]. On the other hand, accidents involving *L. obliqua* specimens have been reported since 1989 in southern Brazil in Santa Catarina, Rio Grande do Sul, and Paraná, also affecting states in the southeast region [10,15,16,24,25,26].

In Brazil, accident notifications are registered and informed by the Brazilian Ministry of Health through the Information System for Notification of Diseases (SINAN). Data from SINAN between 2007 and 2017 [10] indicate that most cases of accidents involving caterpillars occurred in southern and southeast states during the warm and rainy season [9,10], representing ideal conditions for the hatching of eggs and subsequent development of larvae. A study performed with 105 patients in the State of Santa Catarina (between December 1998 and June 2000), showed that most accidents occur in rural areas (85%), during work activities (55%) [8]. Envenoming by *L. obliqua* caterpillars is considered a public health problem in southern Brazil [10,27,28]. The relevance of the accident is due not only to the increase in the number of accidents, but also to the expansion of the caterpillar population to other areas of the country and to the hemorrhagic syndrome that affects the victims.

## 3. Clinical Manifestations and Complications

Immediately after contact with the caterpillar bristles (Figure 2b), an urticating dermatitis occurs, accompanied by pain and swelling. Some general and nonspecific manifestations may appear later, such as holocranial headache, general malaise, nausea and vomiting, anxiety, myalgia, and, less frequently, abdominal pain, hypothermia, and hypotension. After a period that can vary from 1 to 48 h, blood dyscrasia appears, accompanied or not by hemorrhagic manifestations that usually appear 8 to 72 h after contact [7,8,27,28,29,30]. Ecchymoses can be found, which can reach extensive hemorrhagic dysfunctions, hematomas caused by trauma or healed lesions, hemorrhages from mucosal cavities (gingivorrhagia, epistaxis, hematemesis, and enterorrhagia), macroscopic hematuria, bleeding from recent wounds, intraarticular, abdominal (intra- and extraperitoneal), pulmonary, and glandular (thyroid, salivary glands) hemorrhages, and intraparenchymal cerebral hemorrhage [15,16,17,18,19,27,28] (Figure 2b). Lonomism is the term used to designate the severe hemorrhagic disease related to *Lonomia* accidents [31].

The main complication of *L. obliqua* envenomation is acute renal failure, which can occur in up to 12% of the cases, being frequent in patients over 45 years old and in those with heavy bleeding [24,28,29,30,32]. Moreover, some deaths related to hemorrhage and renal failure have been reported [9,10,15,16,29]. However, the early diagnosis and proper treatment with ALS within 12 h of contact can prevent severe coagulopathy and hemorrhage events [8,15,19,33,34,35].

Considering that caterpillars have gregarious habits, the severity of symptoms may be influenced by the number of caterpillars crushed on contact, the extent of the exposed body area, the depth of wound, and the amount of venom inoculated [31].

According to the intensity of the hemostatic disturbances [8,27,28], accidents can be classified as follows:

(a) Mild: patient with local envenomation and without coagulation changes or bleeding within 12 h after the accident, confirmed with the identification of the agent.
(b) Moderate: patient with local manifestations, alterations in global coagulation tests, or hemorrhagic manifestations in the skin and/or mucous membranes (gingivorrhagia, ecchymosis, hematoma), and hematuria, without hemodynamic alterations (hypotension, tachycardia, or shock) (see Figure 2b).
(c) Severe: patient with impaired coagulation, hemorrhagic manifestations in the viscera (hematemesis, hypermenorrhagia, pulmonary bleeding, and intracranial hemorrhage), hemodynamic changes, and/or failure of multiple organs or systems.

## 4. The Importance of Butantan Institute in Antilonomic Serum Treatment

The hemostatic disturbances observed in the envenoming by *L. obliqua* caterpillars result in a consumption coagulopathy (resembling a DIC) and secondary fibrinolysis, which can lead to the hemorrhagic syndrome [8,19,28,36]. Treatments with antifibrinolytic drugs such as aprotinin and ε-aminocaproic acid associated with whole blood, fresh-frozen plasma, or cryoprecipitates were initially used to treat patients; however, rather than reverting symptoms, the treatment exacerbated them [3,6,7,14,16,28]. In 1996, Da Silva and collaborators [3] developed the ALS from horses immunized with four doses of *Lonomia obliqua* bristle extract (LOCBE), producing antibodies capable of neutralizing the components responsible for typical noncoagulated blood induced by contact with *Lonomia* caterpillars in rats. The developed antivenom is composed of specific immunoglobulin F(ab’)2 fragments purified from horse plasma [4].

Clinical studies showed that hemostasis alteration in this kind of accident could be severe within the first 6 h, with intense fibrinogen reduction [8,19]. The patients with the occurrence of abnormal coagulation in screening tests, such as thrombin time (TT), fibrinogen (Fg) concentration, and whole-blood clotting time (WBCT) or hemorrhage manifestations must be hospitalized to receive treatment with ALS according to the Guidelines of the Ministry of Health of Brazil. After the introduction of ALS therapy, the number of deaths resulting from lonomism was reduced. Currently, ALS is the only specific therapy used to treat victims of *L. obliqua* envenomation and is widely distributed by the Ministry of Health in Brazil. Fibrinolytic agents are not recommended for treatment as the venom mainly contains procoagulant agents [7,8,27,28,36]. The correction of anemia due to blood loss should be instituted through the administration of packed red blood cells [27]. Whole blood or fresh plasma are contraindicated, since intravascular coagulation can be accentuated. The recommended doses of ALS in the treatment, according to severity, are shown in Table 1.

## 5. Blood Coagulation Alterations

One of the main clinical manifestations of *Lonomia* envenoming is the consumption of coagulopathy due to depletion of coagulation factors, as well as a secondary activation of fibrinolysis accompanied by bleeding into the skin or mucosa, as described by Zannin and collaborators [8]. Hemostatic changes were observed in patients who have had contact with larvae of 5 cm or more corresponding to the last larval instars shortly before entering the pupal stage [36].

The hemorrhagic syndrome developed by patients is a consequence of a type of DIC [8,28]. DIC is defined as a pathological syndrome that results in thrombin formation, activation and consumption of some coagulation factors, and fibrin clot formation [37].

According to Zannin and collaborators [8], the global clotting times (thrombin time—TT, prothrombin time—PT, and activated partial thromboplastin time—aPTT) were prolonged in most cases and were related to an intense reduction in plasma fibrinogen. The levels of von Willebrand factor (vWF), Protein S, tissue plasminogen activator (tPA), and urokinase were not altered, while the levels of factors V and VIII and prekallikrein (PK) were reduced, and this reduction can be attributed to consumption suffered in the activation of coagulation. Factors XII, II, and X levels were unchanged. These results indicate that the consumption coagulopathy developed in this envenomation is different from that observed in DIC associated with other clinical conditions, in which these factors are usually reduced [37,38,39]. On the other hand, activation of the contact phase of coagulation is unlikely since factor XII levels were normal, although PK levels were shown to be reduced. Zannin and collaborators [8] suggested that PK could be activated by some component of the venom. A subtle reduction in factor XIII was observed in patients envenomed by *L. obliqua*, different from *L. achelous* envenoming where a drastic reduction in this factor is observed, attributed to a factor that degrades FXIII, present in the hemolymph of *L. achelous*, which was called “Lonomin V” [40]. The generation of fragments 1 and 2 of prothrombin (F1+2) and the thrombin/antithrombin complex (TAT) was also observed, like DIC. Although the generation of F1+2 and TAT confirms the formation of thrombin, the number of platelets was not altered in the blood of the patients. Regarding coagulation inhibitors, there was a marked reduction in the levels of Protein C, and there was no significant consumption of antithrombin (AT), as observed in other cases of DIC. Moreover, the formation of thrombin and the TAT complex was observed mainly in patients with severe coagulopathy. These results suggest that AT activity does not depend on coagulation activation. Thrombocytopenia in patients is rare, and its absence can be explained by the high generation of fibrin degradation products (PDFn) suggested by the extremely high levels of D-dimers (DD) [8,19,41,42]. In patients with high levels of DD, a reduction in proteins involved in the fibrinolytic system such as plasminogen, plasminogen activator inhibitor (PAI), and α2-antiplasmin (α2-AP) was observed. In blood coagulation, DD is generated by the action of plasmin on crosslinked fibrin, while the action of plasmin on fibrinogen is observed by the generation of another class of degradation products (PDFg) [43]. Thus, the high increase in DD in patients envenomed by *L. obliqua* suggests that the observed fibrinolysis is an event secondary to the formation of intravascular fibrin [8,19].

In summary, LOCBE induces consumption coagulopathy, depletion of some coagulation factors and inhibitors, and secondary fibrinolysis. It induces a special form of DIC, different from that observed in other clinical situations such as trauma, neoplasia, and sepsis [8,19,28,37,39].

## 6. Toxins Identified in LOCBE

In vitro studies with LOCBE revealed that it has mainly procoagulant activity [43,44,45,46,47,48]. However, several studies have identified in bristles or hemolymph many biological activities that could be associated with the effects observed in patients such as inflammation, leukocyte migration, degradation of extracellular matrix, or even pain. Table 2 lists the main biological activities, toxins, or transcripts identified in *L. obliqua* tissues and secretions.

Transcriptome and proteome approaches have shown the complexity of LOCBE at the molecular level. However, these approaches did not provide a clear identification of procoagulant molecules or toxins equivalent to well-known coagulation activators [31,48]. The most studied toxins isolated from LOCBE are two procoagulant proteins: the *Lonomia obliqua* Stuart factor activator (Losac) and the *Lonomia obliqua* prothrombin activator protease (Lopap). Both proteins, far from being similar to known clotting factors, were identified as belonging to the hemolin and lipocalin families, respectively [43,47].

### 6.1. Expressed Genes in LOCBE

Strategies based on expressed sequence tags (EST) were commonly used for identifying a large number of genes in species of interest until the development of high-throughput methods [79]. This approach was used to identify and characterize the major transcripts present in LOCBE [51,53]. Reis and collaborators [47] identified and submitted to GenBank in 2004 sequences from 1270 independent clones assembled into 702 clusters of distinct genes and corresponding proteins such as lipocalins, hemolins, serpins, and other proteins (Table 2). The Lopap whole sequence is identified in GenBank by access number AY908986 [47,51].

Aiming to maximize the identification of putative toxins in *L. obliqua* tissues, Veiga and collaborators constructed separate cDNA libraries from both bristle and tegument mRNAs [49]. These libraries correspond to mRNA isolated from *L. obliqua* bristles and from tegument. A catalog for the transcripts from *L. obliqua* structures showed that lipocalin is the most abundant transcript in this genome. Both cDNA libraries of *L. obliqua* contain sequences with homology to lipocalins. A total of 1152 independent clones from the tegument library and 960 from the bristle library were identified as expressed, yielding 938 and 730 sequences, respectively [49].

In the *L. obliqua* bristles library, over 50% of cDNAs code for a lipocalin, followed by kininogen (16.5%), serine proteases (14.7%). and lectin (5.5%) [49]. Concerning the tegument library, the number of clusters found for serpins is the most abundant (25.8%), followed by serine proteases (16.1%), lipocalin (16%). and lectin (12.9%). These gene sequences from both cDNA libraries were applied independently in GenBank. and they are complementary [47,49,51]. Sequence analysis also showed that Lopap is a member of the lipocalin family of proteins, since it presents an identity of 20% to 59% with other lipocalins [47,51]. Lopap has a serine protease-like activity and acts on prothrombin, such as FXa, in the absence of prothrombinase components [47]. Characterization of the transcripts present in LOCBE showed several kinds of components that distinctly take part in the envenoming. However, the exact toxins involved in the envenomation are not entirely clear so far.

### 6.2. Proteomic Analysis of LOCBE

Proteomic analyses of LOCBE are scarce, and studies are focused on specific bioactive molecules related to the hematological disturbances observed during envenomation rather than an in-depth description of the bristles extract protein content [50]. A protein profile of LOCBE analyzed through 2D electrophoresis revealed the presence of 159 to 129 spots under nonreducing or reducing conditions, respectively. Most of the spots were detected at acidic to neutral isoelectric point values (4 < pI > 7) distributed in a wide molecular mass range (<10 to 105 kDa). This complexity was predominantly diminished at low molecular mass range under reducing conditions, suggesting the presence of dimers or oligomers, and its monomers were not retained on the acrylamide gel mesh used. According to Coomassie blue staining and immunogenic potential, 25 spots were submitted to mass spectrometry analysis, and three protein categories were identified: lipocalins (eight spots), cuticle proteins (five spots), and serpin (one spot). Twelve spots were described as unknown proteins; some of them were immunodetected using ALS or anti-Lopap rabbit serum, suggesting the presence of interesting immunogenic molecules to be further investigated [50].

### 6.3. Procoagulant Toxins from LOCBE

Losac is the first factor X activator purified as a monomer of 45 kDa from LOCBE [43,63]. The cloning, heterologous expression, and characterization of recombinant Losac (rLosac) was described by Alvarez-Flores and collaborators [43,48]. rLosac specifically activates factor X in the absence of calcium and phospholipids, although the presence of these cofactors accelerates its activity. Its enzymatic characterization was performed, revealing that this protein has no homology to known procoagulant proteases. Instead, Losac belongs to the hemolin family of proteins, a group of multifunctional proteins exclusively expressed by Lepidoptera order insects involved in several cell interactions, but mainly in immunity [43,80]. The tertiary structure model of rLosac was built through homology modeling using the crystal structure of *H. crecopia* hemolin as a template, and it shares the multidomain structure D1–D4 and its conserved motifs. In addition, the multiple amino-acid sequence alignment of rLosac showed up to 76% identity with other hemolin protein families [43].

Studies were conducted to evaluate its effects on endothelium. rLosac inhibited apoptosis in serum-deprived human umbilical vein endothelial cells (HUVECs) and induced cell proliferation [63]. This reduction in cell death under nutrient deprivation conditions was also observed in mouse cortical neurons and human dermal fibroblasts, showing an effective prevention of reactive oxygen species generation and loss of mitochondrial membrane potential, suggesting an antioxidant activity [60,61,62]. An in vivo experimental model in rats demonstrated that rLosac improved wound healing by increasing the epidermal proliferation, as well as by preserving the extracellular matrix organization through collagen type I, fibronectin, and laminin expression. Thus, rLosac was indicated as a very promising molecule, potentially useful as a bioactive agent to develop new formulations for wound healing [59].

Lopap is a 69 kDa prothrombin activator that shares with LOCBE its role in inflammatory processes and belongs to the lipocalin protein family, being the most abundantly studied isolated toxin from the LOCBE [47,48,51,57]. The recombinant form of Lopap (rLopap) recognizes and hydrolyzes prothrombin, which in turn leads to an active thrombin generation, showing a proteolytic activity similar to native Lopap [47,57]. Lopap displayed a Ca^2+^-activating serine protease activity that was included into the group I of prothrombin activators [36,45,47]; the infusion of native Lopap produced intravascular coagulation and thrombosis in the post capillary vessels of mice [46].

Properties of rLopap were evaluated in an in vivo model of leukocyte–endothelial cell interaction, revealing that rLopap as the native Lopap induced NO production and ICAM-1 expression in both neutrophils and endothelial cells. In addition, it induced antiapoptotic effects mediated by NO production [81]. The study of its effects in human platelets showed that there is not a direct effect on platelet function, since Lopap showed no effect on platelet aggregation induced by collagen ADP or thrombin. On the other hand, Lopap induced the expression of adhesion molecules ICAM-1 and E-selectin of human endothelial cells [56]. In those cells, Lopap promotes survival mechanisms since it induces the release of nitric oxide and prostaglandin I2, along with the release of inflammatory cytokine IL-8 and t-PA. Moreover, synthetic peptides based on lipocalin motif 2, which is found in a primary sequence of Lopap, showed cytoprotective and antiapoptotic activity in vitro and in vivo approaches, suggesting the involvement of that lipocalin motif in cell protection [82,83,84,85]. This knowledge brought new perspectives on the use of these synthetic molecules since they do not exhibit hemostatic functions.

## 7. Effect of LOCBE and Toxins in the Inflammatory Response

Dermatitis and skin reactions such as urticaria are well-known signs after accidental contact with the spines and bristles of venomous lepidopteran caterpillars [1]. Generally, the consequences of these reactions are limited to local inflammation, with no systemic or tissue damage. In the case of the envenomation caused by *L. obliqua*, the process is characterized by triggering an intense inflammatory response in victims followed by coagulation, complement, and kallikrein–kinin systems [36,48,51]. In recent years, several studies have been carried out aiming to clarify and describe the role of the venom-induced inflammatory response in the clinical symptoms characteristic of lonomism.

*L. obliqua* proinflammatory effects are first manifested by pain, burning sensation, edema, and erythema formation [8,19,28]. The first pharmacological studies showed that venom-induced nociception in animal models is largely facilitated by the production of prostaglandins, and later edematogenic symptoms are induced by prostanoids and histamines [48,71]. The kallikrein–kinin system is also involved in the edematogenic and hypotensive responses triggered by the venom. Bohrer and collaborators demonstrated that administration, prior to treatment with LOCBE, of plasma kallikrein inhibitor reduces the volume of venom-induced edema in a mouse paw model [35].

Envenomed patients presented low levels of plasma prekallikrein [8,19], indicating that kallikrein was activated and released into the blood circulation. The kallikrein–kinin system is composed of proteolytic enzymes and their substrates, being able to generate potent vasoactive and proinflammatory molecules that are involved in the control of blood pressure, vascular permeability, vascular smooth muscle cell contraction or relaxation, and pain [86]. One of the common consequences of lonomism is the sudden loss of basic renal functions. Kidneys and urine from envenomed animals were enriched with proteins related to inflammatory stress, tissue damage, oxidative stress, coagulation, complement system activation, and kinin system [87,88]. When simultaneously treated with kallikrein inhibitors and antilonomic serum, envenomed rats showed improvements in renal and vascular function, reducing tubular necrosis and renal inflammation [32]. The mechanism underlying these effects was associated with lowering renal inflammation, with a decrease in proinflammatory cytokines and matrix metalloproteinase expression, reduced tubular degeneration, and protection against oxidative stress.

An increase in the permeability of the endothelium allows greater infiltration of cells of the immune system, effectors, and regulators of acute inflammation into tissues [89]. Increased vascular tissue permeability is a characteristic event of the inflammatory response and can be induced by several proinflammatory and vasoactive substances such as bradykinin, histamine, thrombin, cytokines, prostaglandins, and free radicals [74,90]. Activation of the vascular tissue was observed after a single subcutaneous injection of LOCBE in rats [91]. Envenomed animals demonstrated neutrophilic leukocytosis in several tissues, where their histological sections provided evidence of inflammatory cell infiltrates in the heart, lungs, and kidneys, characterizing a systemic acute inflammatory response induced by the venom [92]. Furthermore, an increase was observed in leukocyte rolling and adhesion of these circulating blood cells to the endothelium of hamster cheek pouch tissue that was previously incubated with low doses of LOCBE [72]. The ability of LOCBE to induce an increase in the permeability of the vasculature and immune cell infiltration may provide a favorable environment for hemorrhages, especially in microvessels in the brain.

Due to the important relationship between inflammation and vasculature, studies were carried out seeking to elucidate the direct effect of LOCBE on vascular tissue. In vitro studies showed that non-hemorrhagic concentrations of LOCBE modify the cytoskeleton dynamics and increase focal adhesion in endothelial cells [72]. Furthermore, low doses of the LOCBE can induce activation of the nuclear transcription factor κB (NF-κB) pathway in these cells [73]. The NF-κB pathway is a critical signaling in several events associated with triggering acute inflammation and immune system cell recruitment [93]. Consequently, LOCBE also induced significant increases in the expression of COX-2, NOS-2, HO-1, MMP-2, and MMP-9, enzymes related to prostaglandin production, oxidative stress, and extracellular matrix degradation [72,74].

Additionally, the LOCBE was also shown to be a potent activator of vascular smooth muscle cells, being able to induce cell chemotaxis, exacerbated proliferation, and production of reactive oxygen species. Smooth muscle cell dysfunction is characterized by increased cell migration and proliferation, events that are amplified by the release of inflammatory mediators [91]. Furthermore, researchers also carried out a broad analysis of the gene expression profile of fibroblasts treated with LOCBE. The results show an upregulation of several proinflammatory mediator genes, such as IL-8, IL-6, and CCL2, as well as the adhesion molecule ICAM-3 and COX-2 [74]. Recently, our group showed a direct effect of LOCBE upon macrophage activation. The LOCBE directly induces THP-1 macrophages to a proinflammatory phenotype by activating NF-κB pathway, leading the cells to release proinflammatory cytokines and chemokines such TNFα, IL-1β, IL-6, IL-8, and CXCL10 [73].

The role of isolated toxins present in the venom triggering inflammatory responses is still to be investigated. In addition to its procoagulant activity, in vivo studies show that injection of a high concentration of recombinant *L. obliqua* prothrombin activator protease (rLopap) in rats promotes neutrophil and monocyte infiltration in pulmonary microcirculation vessels. In HUVECs, rLopap stimulates the increase of IL-8, ICAM-1, and E-selectin, proteins involved in the recruitment of immune cells to the tissue [56,75]. In contrast, there is no evidence that Losac presents proinflammatory activity beyond its cytoprotective and proliferative effects.

Taken together, the evidence indicates that LOCBE can induce a local acute inflammatory response that can evolve into a systemic response. Many studies have characterized the role of the kinin–kallikrein system and the liberation of other proinflammatory mediators by the affected tissues, related to several clinical stomps. The isolated effect of the toxins present in the LOCBE, such as Lopap, and their roles in the activation of prekallikrein, the ability to directly induce cell responses, and the molecular mechanism underlying these effects still need to be clarified.

## 8. Innovation in Toxinology: Research Centers and New Molecule Development

### 8.1. Research Centers Specialized in Toxinology

Created in 1962, the State of São Paulo Research Foundation (FAPESP) has robust programs of fellowships and research aiming to promote scientific research in the state of São Paulo. Focusing on the production of multidisciplinary scientific knowledge with high-impact science, in 2000, FAPESP started a program to create Research, Innovation, and Dissemination Centers (RIDC). The “Center for Applied Toxinology (CAT)”, dedicated to studying animal and microbial toxins, based at Butantan Institute in São Paulo, Brazil was one of the 17 RIDC selected projects (https://fapesp.br/cepid/pasta_cepid.pdf?t=1; (accessed on 31 August 2001). The CAT’s scientific findings enrolled in toxin-based drugs affecting the blood clotting, cardiovascular system, pain perception, antiproliferative compounds, and immune suppression resulted in the filing of eight patents, and the technology was transferred to three Brazilian pharmaceutical companies [94].

Motivated by the knowledge and technological innovation resulted from CAT experience along its 10 years, the assembled researchers started another ambitious project and created the “Center of Toxins, Immune Response, and Cell Signaling (CETICS)”, a center of excellence in toxins, immune response, and cell signaling, also based at Butantan Institute and supported by FAPESP (https://bv.fapesp.br/pt/auxilios/58571/cetics-centro-de-toxinas-imuno-resposta-e-sinalizacao-celular/; (accessed on 31 August 2001).

Again, according to the rules of an FAPESP program, Butantan Institute initiated a long-term research program enrolling Research Institutes and companies. Created in the public–private partnership (PPP) model, the “Center of Excellence in New Target Discovery (CENTD)” resulted from a partnership among the Butantan Institute, FAPESP, and GlaxoSmithKline Pharmaceutical (GSK). As a multidisciplinary center inaugurated in 2017, the CENTD focus is to use venoms and secretions to validate new therapeutic targets for inflammatory diseases, such as rheumatoid arthritis, metabolic syndrome, and neurodegenerative diseases. Recently renewed, CENTD has so far achieved eight patents and many scientific publications.

### 8.2. New Molecular Entities for the Study of Anti-Inflammatory Diseases

Lopap is the most studied lipocalin isolated from *L. obliqua*, and the activity of its derived peptides has been the aim of several studies.

Lipocalins are known by their role as protective factors with participation in development, regeneration, and tissue repair [95,96,97]. Moreover, several lipocalins have been involved in the mediation of cell regulation, such as cell growth and differentiation [98,99]. Lipocalins share three main structures: structurally conserved region 1 (SCR1) is the highest conserved region with the 3–10 helix and adjacent strand leading into a loop. SCR2 is formed by two strands, and the loop that connects them varies across proteins; however, the essential features of the loop are retained, despite the unfavorable main chain. Lastly, SCR3 contains a strand with arginine as the last residue and part of the loop linking it to the C-terminal α-helix [100].

Synthetic peptides based on these three conserved motifs found in Lopap—pM1, pM2a/pM2b, and pM3 (the numbers are related to the motif)—were studied to determine their capability to promote cell survival. Motif 2-related peptides showed a cytoprotective activity, in which pM2b was found to be the shortest peptide with similar activity to Lopap, despite not containing the residues supposedly involved in the catalytic activity of Lopap. This peptide triggers cell survival of neutrophils and endothelial cells via nitric oxide [82]. The effect of peptide pM2b was also evaluated in the modulation of extracellular matrix (ECM) proteins using both in vitro and in vivo approaches and showed an increased production of ECM, as well as a modulation of mediators involved in apoptosis, anti-apoptosis, and proliferation in human fibroblasts. Additionally, an increased production of collagen was observed in vivo [83]. Peptide pM2b also displayed modulating effects in an in vivo model of wound healing. Furthermore, the increased production of collagen, glycosaminoglycans, and metalloproteinases along with improvement of wound closure was observed [84]. Those studies suggest an involvement of motif 2 in cell protection. Moreover, using a peptide mapping approach and tertiary structure comparison, a lipocalin sequence signature able to modulate cell survival was identified [82]. A computational analysis of the peptide sequence signature YAIGYSC, later called pM2c, revealed great similarity with antiapoptotic lipocalins [85]. Alignment of the Lopap sequence with other lipocalins showed that this peptide sequence was highly conserved, and yet few different patterns could be observed. Ten aligned peptides, along with pM2c, were modeled in 3D structures, followed by the analysis of molecular descriptors. This study showed that, even with the amino-acid modifications, the calculated molecular properties were generally maintained, especially the molecular shape and electronic density distribution, highlighting the importance of these two properties for molecular recognition process, while the lipophilicity was more related to the pharmacokinetic profile, validating the lipocalin sequence signature previously reported [85].

Other peptides have been isolated from Lopap (Table 3). The peptide CNF011.05D, along with three peptides comprising an amino-acid sequence of at least 70% identity, was described and patented for being capable of stimulating the production of ECM proteins in human fibroblast cells (Patent No. P4US88374OB2 in the USA and No. EP2245149B1 in Europe). The ECM proteins evaluated were fibronectin, tenascin, procollagen, and collagen. In addition, it was demonstrated that this peptide induced the production of nitric oxide, and a wound healing model in rats and pigs showed a faster rate of reduced wound size.

Regarding Losac, the structural analysis revealed that it shares the conserved motifs and immunoglobulin-like domains scattered throughout their four domains (D1 to D4) with hemolins from different Lepidoptera species [43]: *N*-glycosylation and protein kinase C in D3 and cAMP/cGMP in D2; furthermore, the conserved Lys–Gly–Asp (KDG) motif is found in domains D1 and D3 [101]. The KDG motif is related to cell adhesion, and previous studies in hemocytes revealed that hemolin could be involved in cell differentiation or regeneration of wounded tissue, acting as a cell attachment component, suggesting its participation in cellular immune response and morphogenesis [102,103]. This also resembles the function of cell adhesion molecules such as selectins and cadherins; they are important in lymphocyte homing during the inflammatory response and morphogenesis, as well as in the development of neural systems [103,104,105].

Despite advances involving the pathogenesis of inflammatory diseases and new therapeutic strategies, the progression of inflammatory diseases has become a public health problem in recent years, mainly due to new viruses and resistant bacteria [106], along with inflammatory diseases related to aging [107]. Therefore, studies on new molecular targets are needed for development of more effective drugs and therapeutic approaches that can benefit the health and quality of life of patients. Accordingly, allied with the promising results from *Lonomia* peptides, several other peptides were engineered and assayed in several in vitro models of inflammatory diseases, such as arthritis. Some of these peptides had anti-inflammatory effects on several cellular models developed at CENTD (Table 3), reversing the expression of molecules associated with the inflammatory mechanism of cells, in addition to decreasing pain markers in the neuron model and inducing regenerative proteins in different cell models. Those peptides were chosen as a powerful tool for the discovery of new inflammatory targets against arthritis, associating omics approaches with the developed cell models and LOCBE.

## 9. Concluding Remarks

The hemorrhagic syndrome is one of the most serious complications in patients who have contact with the *L. obliqua* caterpillar bristles. After the burst of accidents with hemorrhagic manifestations in 1989 in the states of south and southeast Brazil, many efforts were put into finding a solution to the public health problem that reached alarming proportions. Fortunately, the treatment with ALS produced at the Butantan Institute drastically reduced the consequences of the envenoming. All these efforts have resulted in substantial knowledge about the pathophysiology of the envenoming and the toxins contained in the venom.

Over the years, research has diversified, addressing not only the hematological alterations of the venom but also the role of toxins in each observed manifestation (Table 2). New molecules identified demonstrate peculiarities, such as hemolin and lipocalin structures with enzymatic or cellular activities that have never previously been reported for these types of proteins. This opened new perspectives for the use of those molecules in several applications, as diagnostic agents, for example, to detect dysprothrombinemias, as in the case of Lopap, which resulted in several patents granted, or as therapeutic agents for use in defibrinogenating and antithrombotic therapy (Lopap) or for promoting wound healing (Lopap- and Losac-derived peptides) (Table 3).

The creation of multidisciplinary research centers specialized in the study of animal envenomation and animal toxins promoted the interaction of partnerships with the pharmaceutical industry, e.g., CENTD, taking the study of molecules derived from venoms and toxins to another level, favoring the development and innovation of new molecules. High-throughput screening technologies for new drugs or target discovery are extensively used at CENTD using toxin-derived peptides as tools for identifying new targets for inflammatory diseases.

Therefore, it is clear that the efforts of distinct groups at Butantan Institute have not only contributed to accumulated knowledge about the toxinology of *Lonomia*, but also created the basis for the development of ALS, essential in reducing the deaths due to lononism, and prompted the institute to develop innovative initiatives with toxin-derived peptides.

## Figures and Tables

**Table 1:** Classification of the severity of accidents by *L. obliqua* and therapeutic guidance according to the Brazilian Ministry of Health [27,28].

Manifestations of Severity	Clinical Local Picture	Coagulation Time	Bleeding	Treatment
Mild	Present	Normal	Absent	No ALS required
Moderate	Present or absent	Altered	Absent or present in skin/mucous membranes	Serotherapy: 5 ampoules of ALS
Severe	Present or absent	Altered	Present in viscera. Life-threatening	Serotherapy: 10 ampoules of ALS

**Table 2:** Complexity of *L. obliqua* caterpillar venom and secretions.

Toxin/Biological Activity	Source	Method of Detection/Characteristic	References
Lipocalin	LOCBE Hemolymph	Edman sequencing cDNA library More abundant	[47,49]
	LOCBE	Proteome	[50]
	Tegument Cryosecretion	Edman sequencing	[49]
Hemolin	LOCBE	Edman sequencing cDNA library	[47,49]
Serpin	LOCBE	Edman sequencing cDNA library	[49,51]
		Proteome	[50]
	Tegument	Edman sequencing cDNA library	[49]
	Hemolymph Cryosecretion	Edman sequencing	[49]
Kininogen	LOCBE	cDNA library	[49,51]
Trypsin	LOCBE	Edman sequencing	[49,51]
Lectin	LOCBE	Edman sequencing cDNA library	[49,51]
	Tegument	cDNA library	[49]
Transferrin	LOCBE Tegument Hemolymph Cryosecretion	Edman sequencing	[49]
Laminin	LOCBE	Edman sequencing	[49]
Protease inhibitor	Hemolymph	Edman sequencing	[49]
	Tegument	cDNA Library	[49]
Serine proteases	LOCBE	cDNA Library	[49]
Phospholipase A2 (PLA-2)/hemolytic activity	LOCBE	cDNA Library	[49,51]
		Purified protein with Indirect hemolytic activity	[52,53,54,55]
	Tegument	cDNA Library	[49]
Lopap	LOCBE	Proteoma cDNA library Native and recombinant protein Lipocalin-like Prothrombin activator Cytoprotector	[45,46,47,56,57]
Bilin-binding proteins (BBP)	LOCBE	Recombinant Lipocalin Similar to Lopap No prothrombinase activity	[58]
Losac	LOCBE	Native and recombinant protein Hemolin-like Factor X activator Neuroprotection Antiapoptotic	[59,60,61,62,63]
Factor Xa-like	LOCBE	Sequence similar to Lopap Enzymatic activity on S-2222 chromogenic substrate	[64]
Lonofibrase	Hemolymph	Fibrinogenolytic activity	[48,65]
Lonoglyases	LOCBE	Hyaluronidase Degradation of ECM	[66]
Antiapoptotic/proliferative	LOCBE	Activity on several cell cultures	[56,60,62,63,67]
	Hemolymph	Activity on *Spodoptera frugiperda* (Sf-9)	[68]
Antiviral	Hemolymph	Recombinant protein Effect of several virus	[69,70]
Nociceptive and edematogenic	LOCBE	Prostaglandins favors nociception Kallikrein inhibitor or bradykinin B2 receptor antagonists reduces the edema and hypotension	[35,71]
Kallikrein–kinin system activation	LOCBE	In vitro and in vivo studies showed activation of kinin system	[32,35,71]
Proinflammatory response	LOCBE	Proinflammatory phenotype in macrophages and endothelial cells NF-κB pathway activation	[72,73,74]
Modulation of cell adhesion/cytoskeleton dynamics	LOCBE	Changes in cell–ECM interaction	[74,75]
Platelet adhesion and aggregation	LOCBE	Platelet aggregation in vitro inhibited by PLA-2 inhibitors	[76,77,78]

**Table 3:** Patents applied and granted regarding *Lonomia* toxins and derived peptides.

Molecule	Patent Number	Claim
Lopap	INPI (PI0200269-8, Brazil, 26 July 2016).	Lopap obtention process
	WO03/070746 (WIPO)	
	AU2003208190 (Australia)	
	CAN2,471,410 (Canada)	
	EP1482969 (German)	
	JP2003-569653 (Japan)	
	MX04007344 (Mexico)	
	US10/501,238 (USA)	
P4	INPI (PCT/IB2009/05023, Brazil, 21 July 2010)	Tissue remodeling and tissue repair
	101965398B, 2015 (China)	
	JP5777886B2, 2015 (Japan)	
	EP2245149B1, 2015 (German)	
	US8,883,740 B2, 2014 (USA)	
Losac-derived peptides	INPI (BR10201901866, Brazil, 9 September 2019)	Anti-inflammatory agents

## Data Availability

The data presented in this study are available on request from the corresponding author.
